# Management of OSAS: The ELIBA® Device Can Help the Patient?

**DOI:** 10.1155/2020/9873761

**Published:** 2020-03-04

**Authors:** Eleonora Ortu, Davide Pietropaoli, Alessandra Mummolo, IAPNOR, Mario Giannoni, Annalisa Monaco

**Affiliations:** ^1^MeSVA Department, Division of Dentistry, University of L'Aquila, P.le Salvatore Tommasi, 67100 L'Aquila, Italy; ^2^I. A.P.N.O.R.–International Academy of Posture and Neuromyofascial Occlusion Research, Viale Gino Moretti 37, 63074 San Benedetto del Tronto, AP, Italy

## Abstract

Obstructive sleep apnea syndrome (OSAS) is one of the most challenging diseases to treat in medicine. Here, the authors describe a case of OSAS treated with a lingual elevator of Balercia (ELIBA®). The patient, a forty-five-year-old Caucasian male, had a chief complaint of numerous episodes of nocturnal apnea. After several visits with specialists, a polysomnographic examination was performed, in which the patient's apnea hypopnea index (AHI) was 30.4, and a lingual elevator was prescribed. The lingual elevator helped to keep the patient's tongue in the correct position and prevented the tongue from reverting back to the soft tissue spaces in the mouth. After six months of treatment with the lingual elevator and dietary adjustments, the patient's AHI decreased to 11.6. This simple yet customizable approach is a comfortable and easy option for patients to reduce night apnea episodes.

## 1. Introduction

OSAS is characterized by repeated episodes of complete, partial, and/or prolonged obstruction of the upper airway during sleep, normally associated with a reduction in blood oxygen saturation. It occurs in all ages and it is certainly more common in males [1]. Patients present a high number of apneas during the night and complain of excessive sleepiness during the day. Other common symptoms are headache upon waking up, snoring reported by the partner as intermittent (because interrupted by apnea), reduced memory capacity, reduced ability to concentrate, hypertension, and dry mouth upon waking up [2, 3]. The airways can be blocked in several ways. For example, a large tongue, associated with normal muscle relaxation and consequent soft tissue collapse that occurs during sleep, may be one of the causes of apnea [3–5]. Further, overweight or obese people have a predisposition for apnea; symptom severity often increases with increased body weight. Another major risk factor for apnea is nasopharyngeal abnormalities that reduce the upper airway diameter. Though generalized narrowing is common in adults and in children, anatomopathological anomalies such as adenotonsillar hypertrophy can increase the risk of apnea, and deviations of the nasal septum are often observed [6]. A fundamental premise for the treatment of OSAS is an accurate and multidisciplinary diagnosis: the reference centers for sleep medicine usually have a team that includes, in addition to the sleep doctor (specialist in pneumology or neurology) who coordinates activities, other specialists (maxillofacial specialist, orthodontist, otolaryngologist, and nutritionist) who each deal with their own skills to best treat this disease [3, 7]. To reach a correct diagnosis, the clinical work-up requires the association of comprehensive clinical evaluation (anamnesis, physical examination) and nocturnal polysomnography [8]. The last one uses a simultaneous recording of several physiological parameters during the night, using a polysomnograph. Normally, during the test, two or more electroencephalography channels, various electromyographic channels, chest and abdomen movements, oronasal flow, and oxygen saturation in the blood are recorded [9]. There is a variety of treatments for obstructive sleep apnea syndrome, which depends on the medical history of the individual, the severity of the disorder and, mainly, the specific cause of the obstruction. Although there are various oral devices to treat OSAS, many of these devices are cumbersome for patients to use at night because they act to advance the position of the jaw [10–12].

## 2. Case Report

A forty-five-year-old male Caucasian patient had a chief complaint of numerous episodes of nocturnal apnea and daytime sleepiness. The patient had no history of diabetes and asthma. The dental history was insignificant. The patient's body mass index was 29 (overweight), and he was being treated by a nutritionist to improve alimentation and lose weight [13]. The patient's arterial blood pressure was 120/80 mmHg, his pulse was 95 beats/min, and his respiratory rate was 20 breaths/min. The results of the laboratory (i.e., complete blood count, hematocrit, mean corpuscular value, erythrocyte sedimentation rate, and lipid panel) examinations were normal. Chest X-ray, respiratory function tests, and bronchoscopic test results were also normal. The upper airway appeared normal in ear-nose-throat and laryngoscopy examinations. The patient's neck and tongue muscles were enlarged. Written consent was obtained from the patient.

**Figure 1:**
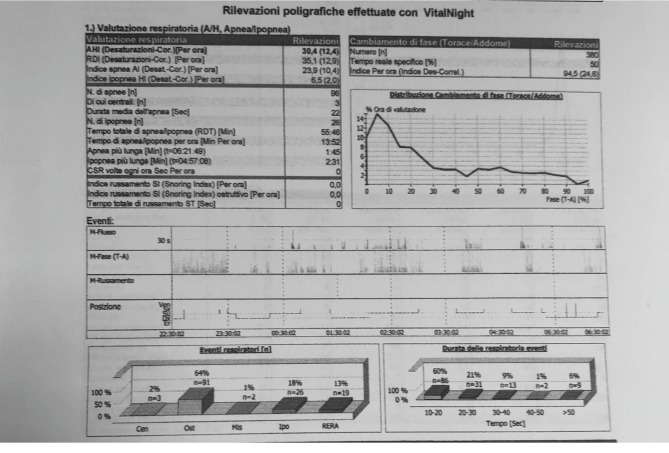
Polysomnographic report before treatment with the ELIBA® device. The apnea hypopnea index (AHI) was 30.4.

Upon polysomnographic examination, the subject was diagnosed with positional preference obstructive sleep apnea syndrome (OSAS), with 70% of episodes occurring while the patient was in the supine position (Figure 1). During the test, he had 96 apneas, 3 of which were central. The patient's apnea hypopnea index (AHI) was 30.4, a value that meets the criterion for severe sleep apnea [14, 15]. Treatment options for reduction of the OSAS, including a lingual elevator by Balercia (ELIBA® device that properly spaces the orolingual space for better breathing) and continuous positive airway pressure (CPAP) therapy, were discussed with the patient. Because the patient refused CPAP therapy due to discomfort, the patient was prescribed an ELIBA®, which is a small, comfortable to wear device that does not change the position of the jaw.

**Figure 2:**
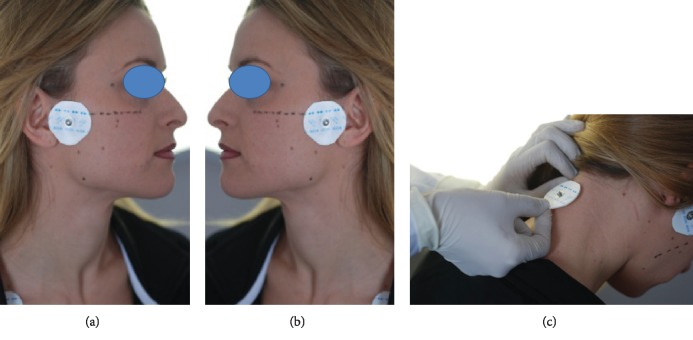
Application of TENS electrodes. (a, b) The two TENS electrodes are placed bilaterally over the cutaneous projection of the fifth pair of cranial nerves notch, which is located between the coronoid and condylar processes. (c) A third grounding electrode is placed in the center of the back of the neck.

**Figure 3:**
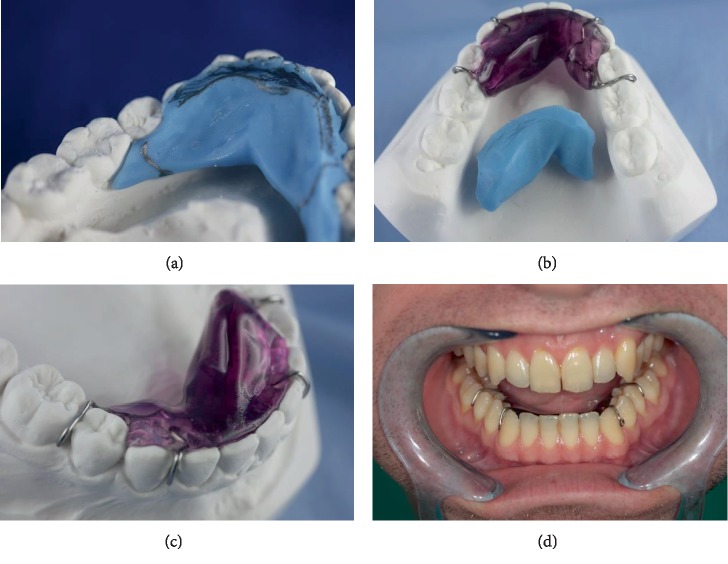
ELIBA® construction phases and oral aspect of the device. (a) Marking of the lateral/distal borders of the ELIBA® device, as specified by the clinician. (b) The ELIBA® appliance on the plastic cast and impression of the ELIBA® device. (c) Enlargement of the device ELIBA® applied on the plastic cast with hooks in front area in cobalt chrome. (d) Intraoral views of the ELIBA® appliance fitted to the oral cavity of the patient.

Briefly, dental stone models of the dental arches and Myoprint impressions of the physiological free orolingual space were made using Bosworth Sapphire acrylic resin (custom acrylic material; Bosworth Company) under ultra-low-frequency-transcutaneous nervous stimulation (ULF-TENS) [16–20]. The low-frequency neurostimulator (J5 Myomonitor® TENS Unit, Myotronics-Noromed Inc., Tukwila, WA) generated a repetitive, synchronous, and bilateral stimulus through pulsed currents delivered at 1.5 s intervals and a frequency of 0.66 Hz. The two TENS electrodes (Myotrode SG Electrodes®, Myotronics-Noromed Inc., Tukwila, WA) were then placed bilaterally over the cutaneous projection of the fifth pair of cranial nerves notch, which was located between the coronoid and condylar processes. This location was determined by manual palpation of the zone anterior to the tragus (Figures 2(a) and 2(b)). A third grounding electrode was placed in the center of the back of the neck [21, 22] (Figure 2(c)). Central nervous system stimulation was produced by sensory stimulation of cranial nerves V and VII with low-frequency TENS [18]. Typically, low-frequency ULF-TENS is applied to bring the mandible in a neutral position that can obviate the need for occlusal adjustments [19]. Once the threshold of sensory stimulus is identified, identified by the sensation of tingling at the level of the electrodes, the stimulus is administered with the ULF-TENS. The patient is asked to rest the apex of the tongue against the back incisive papilla and keep it relaxed in this position for the duration of the procedure. When the resin reached a solid but elastic consistency, before complete hardening, it was extracted from the oral cavity and inserted into the master model to complete its hardening. The clinician indicated to the dental laboratory (enabled to manufacture) the distal lateral limits for the construction of the artifact and the most suitable retentive devices for the stabilization of ELIBA®. ELIBA® must be totally passive and must not touch the oral mucosa [4–9, 19, 23–27] (Figures 3(a)–3(d)).

**Figure 4:**
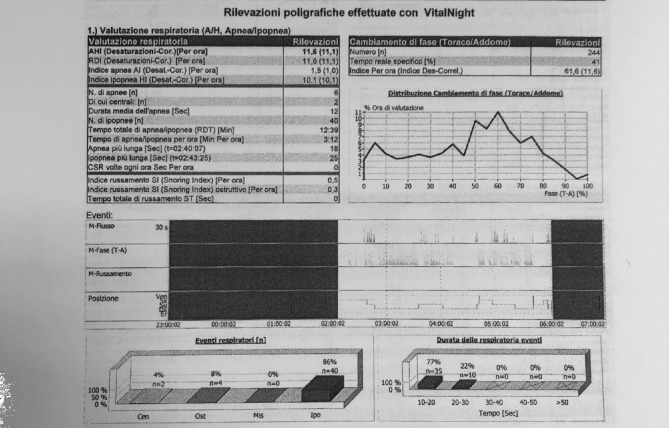
Polysomnographic report after treatment with the ELIBA® device. The apnea hypopnea index (AHI) decreased to 11.6.

After six months of using the ELIBA® device, the patient's apnea improved, and the second polysomnographic examination revealed only six apnea episodes, two of which were central. The patient's AHI decreased to 11.6 after implementation of the ELIBA® device (Figure 4). Moreover, the patient's overall health improved owing to dietary adjustments. He lost ten kilograms, and his body mass index decreased to 25. The patient appeared satisfied with the results of the ELIBA® and dietary adjustments.

## 3. Discussion

The patient described in this case was treated with an ELIBA® device, in strict accordance with the principles of neuromyofascial occlusal dynamics. The ELIBA®, which was designed to treat atypical swallowing and yields better patient compliance than those prescribed CPAP, modifies the position of the tongue by acting on the inner component of the trigeminal system and partly on the autonomic nervous system. ELIBA® seizes the patient's physiological free orolingual space, defined anteriorly and laterally by the lower jaw, inferiorly by the tongue floor, and superiorly by the ventral surface of the tongue. The Medium Longitudinal File, which allows a direct connection between the various cranial nerves, can provide a way to link the upper spinal cord to the mesencephalus. The ELIBA® works by way of proprioception and interception, in which it stimulates the trigeminal system and, consequently, activates the oculomotor system to help maintain appropriate tongue position and movement [28]. Contact of the tongue apex with its designated spot during the night together with perioral muscle-generated centripetal force is indicative of functional improvement. The tongue and neck muscles are related by way of proprioception via a common trunk from the ansa cervicalis through the hypoglossal nerve [29]. Currently, tongue position studies have suggested that it may not be possible to improve OSAS, at least not without difficult-to-use devices, because it is related to a subcortical abnormality, specifically in the ventral part of the rostral brainstem [30, 31].

The ELIBA® is a simple, customizable device that is comfortable for patients to wear and does not have any contraindications in children or adults. In the current case study, ELIBA® implementation combined with dietary changes improved the patient's sleep quality while maintaining patient comfort [32]. The patient was satisfied with the outcome of the ELIBA®. The authors hope to treat further patients with this approach to understand more about its advantages and possible limitations.
